# Which Factors Were Related to the Number of COVID-19 Cases in the 2022/2023 Season Compared to the 2021/2022 Season in Europe?

**DOI:** 10.3390/jcm12134517

**Published:** 2023-07-06

**Authors:** Marharyta Sobczak, Rafał Pawliczak

**Affiliations:** Department of Immunopathology, Division of Biomedical Science, Faculty of Medicine, Medical University of Lodz, 90-752 Lodz, Poland

**Keywords:** SARS-CoV-2, COVID-19, vaccination, omicron, multiple factor analysis

## Abstract

The number of COVID-19 cases was greater in early autumn 2022 in contrast to in autumn 2021. Therefore, we decided to examine the factors that may have affected differences in the number of COVID-19 cases between the time periods 2021/2022 and 2022/2023 with consideration of the occurrence of influenza. In this cross-sectional study, we conducted a multiple factor analysis using data from publicly available databases for weeks 35–14 in 2022/2023 and 2021/2022 for Austria, Germany, Greece, Italy, and Slovenia. In the 2021/2022 season, the analyzed countries had similar profiles and were characterized by restrictions, health system policies, and SARS-CoV-2 variants, such as Alpha, Beta, Delta, Kappa, Eta, as well as Omicron sublineages (BA.1, BA.2), which were positively correlated with the number of new cases of COVID-19 per million people. However, in the 2022/2023 season, the analyzed countries were described by groups of variables corresponding to vaccination, influenza, the number of flights, and the Omicron SARS-CoV-2 subvariant. In summary, crucial factors correlated with the increasing of number of COVID-19 cases in the 2021/2022 season were the presence of dominant SARS-CoV-2 variants as well as the lifting of restrictions and strict health system policies.

## 1. Introduction

Since December 2019, when the first case of coronavirus disease 2019 (COVID-19) was reported, the COVID-19 pandemic has impacted the entire world. The pandemic has not only negatively affected public health and the international economy but has also influenced social and personal aspects of life, leading, for example, to an increase in domestic violence and negative changes in dietary habits [1,2]. In response to the pandemic, governments have implemented different preventive measures, such as lockdowns, mandatory face mask wearing, and social distancing, although effective vaccines could have been more potent for COVID-19 prevention [2]. Many of the restrictions led to social outrage directed towards governments, especially school and workplace closures, restrictions in movement, and gatherings [3]. Nevertheless, interventions such as banning people from public gatherings (workplaces, pubs, etc.) were pointed out as being the most effective [4]. 

Even though, as of 30 April 2023, around 13 billion of doses of COVID-19 vaccines had been administrated worldwide [5], the number of new infections of COVID-19 tends to fluctuate seasonally. In our previous study [6], we observed several variables that shaped the dynamics of COVID-19 infections in summer 2021 and summer 2022 in five selected countries. Both periods were characterized by period-specific variables for summer 2021, such as dominant COVID-19 variants and COVID-19 restrictions, whereas for summer 2022, the important factors were the number of vaccinated individuals, the number of distributed booster doses, and the presence of the Omicron variant. 

Interestingly, the COVID-19 pandemic is not the first pandemic to occur in the 21st century. In 2009, the influenza A H1N1 pandemic was identified by the World Health Organization (WHO). There are similar features between these two pandemics, such as the type of virus, enveloped RNA viruses, which is capable of frequent mutations as well as high transmissibility. Both viruses can cause respiratory diseases and may be transmitted by oral and nasal droplets and indirect contact. Moreover, those diseases have similar symptoms, such as fever and cough with sore throat and myalgia [7]. However, in contrast to the influenza virus, SARS-CoV-2 induces different types of immune responses, such as the disbalanced polyfunctional inflammatory response, which was described in a study conducted in patients with COVID-19 and influenza A(H1N1) [8]. The influenza virus consists of three compartments, the envelope, matrix proteins, and core. There are several types of this virus: influenza A, B, C, and D, of which only influenza A, B, and C can infect humans [9]. Different factors, such as values of the total rainfall depth, temperature, and amount of sunlight may affect the spread of seasonal influenza. Therefore, in different countries, epidemics of influenza have different characteristics. For example, in tropical regions, irregular epidemics occur throughout the whole year, but in temperate regions, epidemics have seasonal characteristics and mostly occur in winter. Each year, this virus may cause a large number of infections with a large number of deaths [10]. In turn, seasonal epidemics caused by influenza or respiratory syncytial virus (RSV) provoke morbidity and mortality in children. However, during the COVID-19 pandemic, the detection rates of these viruses decreased [11]. Moreover, several studies have shown the occurrence of co-infections with both viruses, which may increase the severity of symptoms. Despite this, co-infection with influenza virus may have a protective effect in COVID-19 patients [10]. Therefore, in this cross-sectional study, we decided to conduct an analysis of variables that may affect differences in the number of COVID-19 cases between the 2021/2022 and 2022/2023 time periods in five chosen countries of the European Union considering the simultaneous occurrence of the influenza season.

## 2. Materials and Methods

### 2.1. Data Search and Extraction

In this study, we decided to continue the analysis from our previous paper; therefore, we included the same set of European Union member-countries [6]. We extracted data from the COVID-19 Vaccine Tracker [12], Oxford COVID-19 Government Response Tracker [13], Our World in Data [14], CoVariants [15], Influenza Surveillance Report of WHO [16], and Eurocontrol [17] between weeks 35 and 14 in 2021/2022 and 2022/2023. 

The data collected and analyzed included continuous variables, such as the number of new cases per million people; the number of cases with different variants of SARS-CoV-2; the number of people with primary courses of vaccination per hundred people; the number of total boosters per hundred people; the number of flights; as well as categorical variables, including schools closing on a scale from 0 to 3; workplaces closing on a scale from 0 to 3; cancelation of public events on a scale from 0 to 2; restrictions on gatherings on a scale from 0 to 4; public transport closures on a scale from 0 to 2; stay at home requirements on a scale from 0 to 3; restrictions on internal movement on a scale from 0 to 2; international travel control on a scale from 0 to 4; testing policies on a scale from 0 to 3; contact tracing on a scale from 0 to 2; facial coverings on a scale from 0 to 4; vaccination policies on a scale from 0 to 5; and protection of elderly people on a scale from 0 to 3. Additionally, we collected data about the number of positive influenza cases from the Influenza Surveillance Report of the WHO [16]. 

Data on the number of cases with different variants of SARS-CoV-2 were collected from CoVariants [15] and calculated using the population count from the Worldometer [18] as the ratio per million. Similarly, the number of people who underwent primary courses of vaccination and the total number of boosters given were collected from the COVID-19 Vaccine Tracker [12] and also calculated as the ratio per hundred people using data from the Worldometer [18]. The number of positive influenza cases was also calculated as the ratio per hundred people using data from the Worldometer [18].

### 2.2. Statistical Analysis

Using collected data, we conducted a multiple factor analysis (MFA), which can be applied for different types of variables (categorical, quantitative, and frequency). The MFA consists of two steps: First, a PCA (principal component analysis) is conducted and each data table, in which sets of variables are collected, is normalized. Additionally, in the second step, these data tables are combined into a joined data table that is analyzed by the PCA [19,20]. We calculated the mean score for the continuous variables and used standardization and centering around zero, while for categorical variables, we used the median. Variables were grouped into new cases, influenza, variants, vaccinations, flights, restrictions, and health system policies as active groups, and years and country names were used as supplementary groups. The MFA analysis was conducted in R (version 4.3.0).

## 3. Results

### 3.1. Comparison of COVID-19 Infections in Analyzed Countries

**Figure 1:**
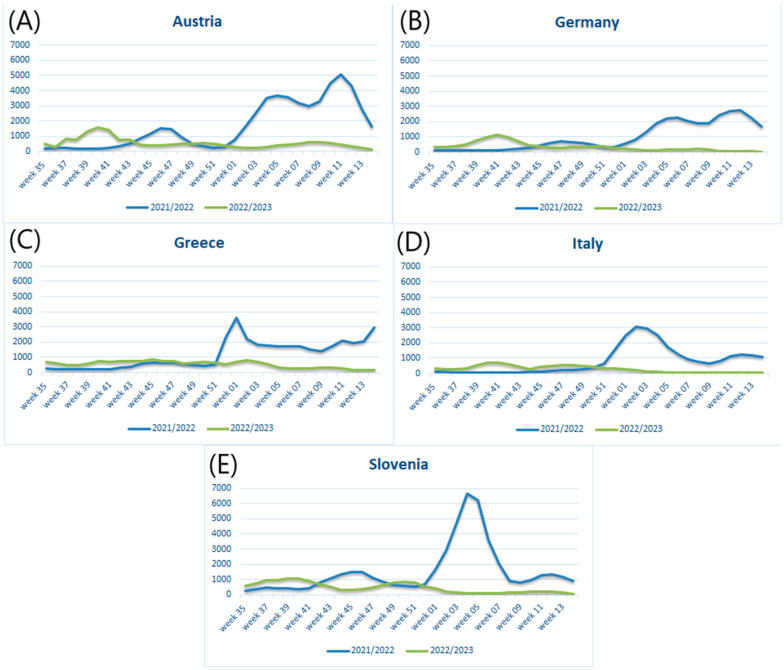
Distribution of COVID-19 cases between weeks 35 and 14 in 2021/2022 and 2022/2023. (**A**) Austria, (**B**) Germany, (**C**) Greece, (**D**) Italy, (**E**) Slovenia.

In the first step, we compared the number of weekly cases of COVID-19 in five analyzed countries: Austria, Germany, Greece, Italy, and Slovenia. As shown in Figure 1A–E, there were differences in the distribution of COVID-19 cases in the 2022/2023 season in comparison to 2021/2022. The peak wave in autumn 2022 was observed around weeks 40–41, while in 2021, it was around weeks 45–47 and later. In the autumn of 2022 in Greece, as well as in the autumn of 2021 in Italy, clear peaks in the number of new infections were not apparent. Later, in the 2022/2023 season, the peak in cases was not observed in all countries, in contrast to the 2021/2022 season.

### 3.2. Definitions of the Contributing to the Dimensions: Dim-1 and Dim-2

**Figure 2:**
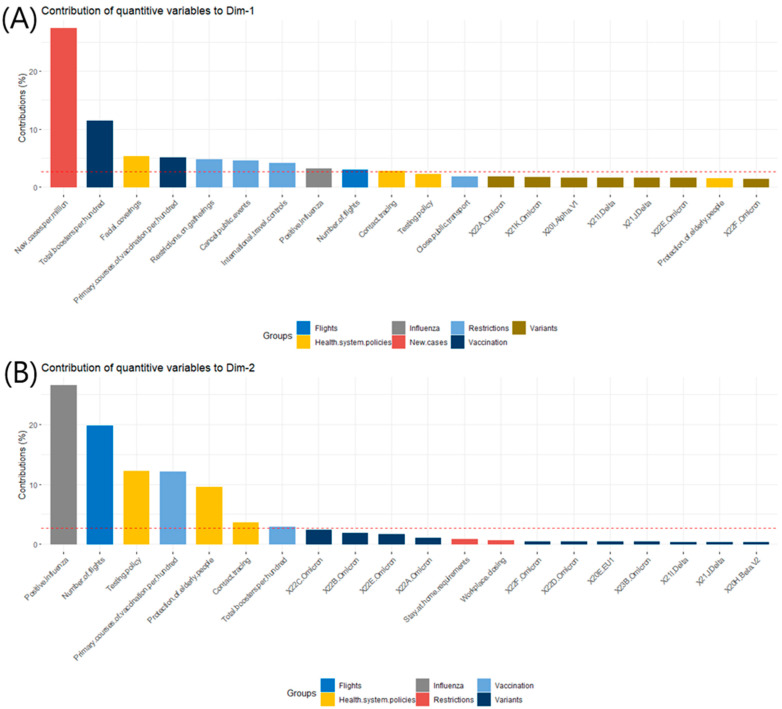
The contributions of quantitative variables to (**A**) dimension 1 (Dim-1) and (**B**) dimension 2 (Dim-2).

The variable ‘new cases per million’ was the variable with the greatest contribution to the definition of dimension 1 (Dim-1), whereas ‘positive influenza per hundred’ was the biggest contributor to dimension 2 (Dim-2), as shown in Figure 2A,B.

### 3.3. The Relationships between Analyzed Variables

**Figure 3:**
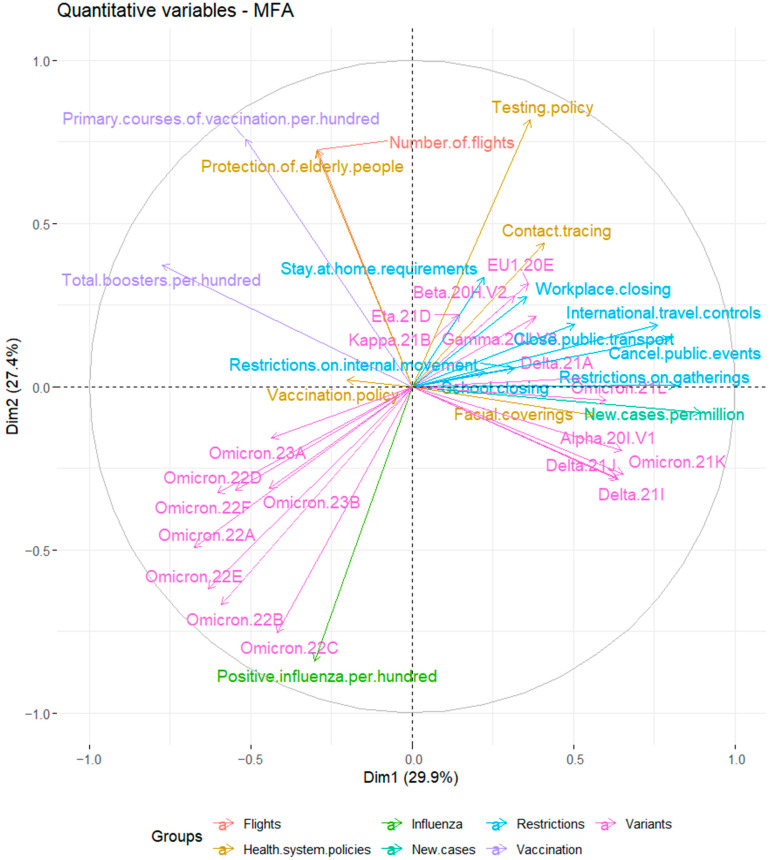
Scatter plot of the analyzed variables.

The reletionships between analyzed variables are shown on a correlation plot in Figure 3. The restrictions and health system policies variable groups, as well as cases of Alpha (B.1.1.7), Beta (B.1.351), Gamma (P.1), Delta (B.1.617.2), Kappa (B.1.617.1), Eta (B.1.525), EU1 (B.1.177), and the Omicron sublineages 21K (BA.1) and 21L (BA.2) were positively correlated with the number of new cases of COVID-19 per million people. The number of positive influenza cases per hundred people was strongly and positively correlated with the Omicron SARS-CoV-2 variants, especially, 22A (BA.4), 22B (BA.5), 22C (BA.2.12.1), 22D (BA.2.75), 22E (BQ.1), 22F (XBB), 23A (XBB.1.5), and 23B (XBB1.16) lineages, whereas it was weakly correlated with the total number of boosters per hundred people.

### 3.4. Comparison of Variable Profiles for Analyzed Countries in Autumn 2021 and 2022

**Figure 4:**
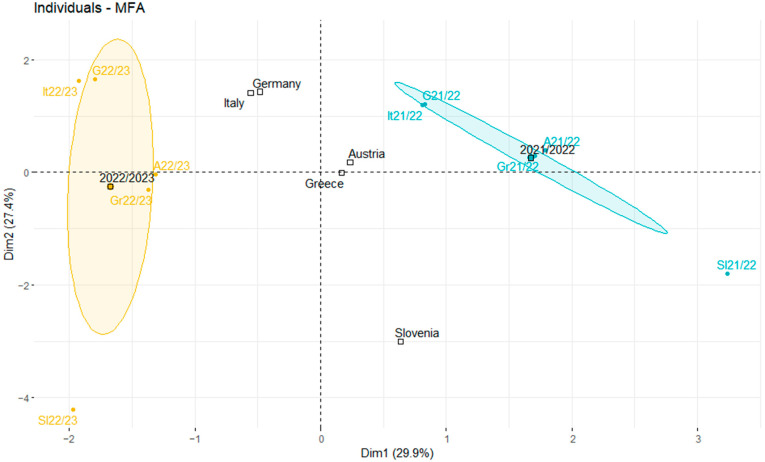
Individual country results for 2021/2022 (teal) and 2022/2023 (yellow). It—Italy, G—Germany, Gr—Greece, A—Austria, Sl—Slovenia.

In the last step, we compared the profiles of the analyzed periods in the 2021/2022 and 2022/2023 seasons. As shown in Figure 4, in the 2021/2022 season, the analyzed countries had similar profiles and were characterized by restrictions, health system policies and SARS-CoV-2 variant groups, while in the 2022/2023 season, the analyzed countries were described by groups of variables, such as vaccination, influenza, the number of flights, and the Omicron SARS-CoV-2 subvariants. 

## 4. Discussion

In our cross-sectional study, we analyzed the influences of variables pertaining to the number of new COVID-19 cases, the number of patients suffering from particular variants of SARS-CoV-2, vaccination, and the severity of restrictions in the 2021/2022 season compared to the same period in 2022/2023 in five chosen counties: Austria, Germany, Greece, Italy, and Slovenia. The 2021/2022 season was mostly characterized by restrictions and infections with the Alpha, Beta, Gamma, Delta, Kappa, Etha, EU1, and Omicron (BA.1 and BA.2) SARS-CoV-2 variants. On the other hand, in the 2022/2023 period, the dominant variables were connected to the presence of Omicron variants, the number of flights, the number of influenza-positive cases, and the number of administered booster doses, as well as the number of primary courses of vaccination. 

As in the 2021/2022 season, some of the COVID-19-related restrictions and healthcare policies were still actively enforced, as they constituted an important factor affecting the number of COVID-19 spread and mortality, which is in line with the results of our analysis. Following the decision of the WHO to declare COVID-19 as a pandemic on 20 March 2020, many European countries implemented a variety of restrictions and health system policies in an attempt to control the spread of the virus. The restrictions included stay-at-home policies, remote work, restrictions on mobility (including travels), the closing of inessential businesses, and so on. Dergiades et al. [21] reported that the strength of the restrictions at the early stages of the pandemic constituted a key factor in slowing the virus’s spread and limiting its mortality. From a timing perspective, some of the limitations, such as closing schools/universities and banning gatherings were more efficient than, e.g., the stay-at-home order [22]. Moreover, travel restrictions were also relatively successful, as a decrease in the number of flights was correlated with COVID-19 mortality in some of the European countries [23]. On the other hand, some of the implemented measures and their levels of severity should be re-evaluated e.g., excessive surface disinfection and mandatory body temperature readings [24]. The overall positive influence of social restrictions on the limitation of SARS-CoV-2 spread is apparent, as lifting these restrictions in September 2021 in Finland resulted in a massive infection spike [25]. Importantly, the COVID-19 pandemic posed a real struggle for many health systems, even in some Western European countries, such as Italy and the UK [26]. The state of medical facilities, as well as the number of trained primary care doctors were crucial factors affecting the effectiveness of particular healthcare systems [27]. 

Moreover, the analyzed periods varied in terms of the dominant variants of SARS-CoV-2. As of 21 April 2023, there were two variants of interest (VOIs)—Omicron sublineages XBB.1.5 (23A) and XBB.1.16 (23B) [28]. The Omicron variant was reported to the WHO for the first time on 24 November 2021 in South Africa [29]. A study conducted in the UK monitored the occurrence of particular COVID-19 variants in January 2022. A total of 99.2% of sequenced patients were infected with Omicron BA.2, while 0.79% were infected with the Delta variant, indicating the exact moment of replacement of Delta with Omicron as the dominant variant [30]. According to the ECDC (European Centre for Disease Prevention and Control) [31], Omicron sublineages (BA.1, BA.2, BA.3, BA.4, BA.5) appeared at the beginning of 2022, and Omicron sublineages (BA.2, BA.4 and BA.5) circulated in the EU/EEA (European Union/ European Economic Area) until the end of 2022. 

Another crucial variable that influenced the difference in the COVID-19 situation between the 2021/2022 and 2022/2023 seasons was the vaccination status, as during the year-long gap between the two periods of interest, vast progress in worldwide vaccination was made. According to the Our World in Data database, on 1 September 2021, 2.16 billion people were fully vaccinated; while on 1 September 2022, the number was 4.92 billion. Similarly, on 1 September, 20.07 million booster doses had been administered worldwide, whereas on the same day in 2022, 2.43 billion booster doses had been administered [32]. Except for the numbers of distributed doses and boosters, progress has been made in terms of responding to new types of COVID-19 viruses [33]. At this point, as the general population has been dealing with SARS-CoV-2 infections for almost 3 years, some level of background immunity has been achieved, and the main effort should be put into the administration of boosters and testing [34]. In the UK, patients who received booster doses had a reduced risk of hospitalization or death due to the COVID-19 in comparison to patients who received only the standard course of vaccination. Moreover, patients who received the mRNA-1273 vaccine booster dose had a lower rate of severe symptoms than patients who were vaccinated with BNT162b2 [35]. Another study reported that the administration of two doses of either the ChAdOx1 nCoV-19 or BNT162b2 vaccine provided limited immunization against the Omicron variant. Although a booster dose of BNT162b2 or mRNA-1273 provided a substantial effect, this diminished over time [36]. In turn, the bivalent Omicron-containing vaccine, mRNA-1273.214, elicited a response not only against the BA.4/5 lineages, but also against the ancestral SARS-CoV-2 [37]. Kohli et al. [38] compared three different vaccination strategies in terms of limiting infections and hospitalizations due to the BA.4/5 subvariant in a 6 month period from September 2022 to February 2023. According to their estimates, administration of the mRNA-1273.214 vaccine could decrease infections by 40% while decreasing hospitalizations by 48%, providing the best efficacy against the Omicron variant. Slightly worse efficacies were expected from mRNA-1273 and mRNA-1273.222, as they were thought to reach reductions in infections of 34% and 18%, as well as reductions of 42% and 25% in hospitalizations, respectively.

The last variable that correlated with the 2022/2023 season was the number of influenza-positive patients. This association resulted from the influenza outbreak in autumn–winter in the 2022/2023 season. In comparison to the same period in 2021/2022, the number of detected cases increased seven-fold. The vast majority of these infections (87%) were caused by the influenza A viruses, A(H3N2) (71%) and A(H1N1)pdm09 (29%), while the remaining percentage of cases (13%) was related to type B viruses of lineage B/Victoria [39]. In order to control the outbreak, the WHO recommended washing hands and wearing well-fitted face masks as well as highlighting the importance of seasonal vaccination [40]. Moreover, simultaneous vaccination against influenza and COVID-19 may be an effective immunization strategy, as shown in clinical trials. For example, in a study of the NVX-CoV2373 vaccine against COVID-19, where around 400 participants were co-injected with the NVX-CoV2373 vaccine and one the two different seasonal influenza vaccines that were approved in the UK for the use in different age groups, it was shown that simultaneous vaccination with influenza vaccines and NVX-CoV2373 vaccine is safe, and the immunogenicity of influenza vaccine is preserved, while the immunogenicity of NVX-CoV2373 slightly decreases [41]. Similar results were shown in a study conducted in the UK that investigated the effects of the ChAdOx1 and BNT162b2 vaccines against COVID-19 and the influenza vaccine that was appropriate to age of the subject. The administration of different combinations of vaccines was safe and preserved the anti-spike immunoglobulin responses to both COVID-19 vaccines [42]. Interestingly, the retrospective analysis showed that influenza vaccination was related to a reduction in positive tests for COVID-19 and also improved the clinical outcomes of COVID-19 patients [43] as well as reducing COVID-19 mortality [44]. 

Our study has a few limitations. First, starting from 20 June 2022, the European Centre for Disease Prevention and Control (ECDC) stopped collecting COVID-19 data, such as the numbers of COVID-19 cases and deaths [45]. Therefore, we used other sources for these data, as described in the ‘Methods’ section. Similarly, the Oxford COVID-19 Government Response Tracker [13] has not published real-time updates since the end of 2022, because there have been no changes in anti-COVID-19 policies in most countries. Therefore, another problem with the data was that some of the data were either missing or reported with a delay. In spite of all this, we compared the set of variables in order to identify factors affecting the difference in the dynamics of COVID-19 spread between the 2021/2022 and 2022/2023 seasons in Austria, Germany, Greece, Italy, and Slovenia

## 5. Conclusions

Our data suggest that the crucial factor correlated with the increasing number of COVID-19 cases in the 2021/2022 season was the presence of dominant SARS-CoV-2 variants, which was correlated with restrictions and health system policies. In the 2022/2023 season, the dominant variables were groups of variables corresponding to vaccination, influenza, the number of flights, and Omicron SARS-CoV-2 subvariants. Additionally, the discovery of new COVID-19 vaccines, such as mRNA-1273.214, gives hope for better immunization, as they are apparently more efficient than previous vaccines.

## Data Availability

Publicly available datasets were analyzed in this study. This data can be found here: [http://www.worldometers.info/ (accessed on 6 May 2023); https://ourworldindata.org (accessed on 6 May 2023); https://www.ecdc.europa.eu/en (accessed on 6 May 2023); https://www.eurocontrol.int (accessed on 6 May 2023); https://www.bsg.ox.ac.uk/ (accessed on 6 May 2023); https://www.who.int/ (accessed on 6 May 2023); https://covariants.org/ (accessed on 6 May 2023)].
